# Assessment of safety and efficacy against *Bordetella pertussis* of a new tetanus-reduced dose diphtheria-acellular pertussis vaccine in a murine model

**DOI:** 10.1186/s12879-017-2369-x

**Published:** 2017-04-04

**Authors:** Hyo Jin Kwon, Seung Beom Han, Bo Ram Kim, Kyu Ri Kang, Dong Ho Huh, Gi Sub Choi, Dong Ho Ahn, Jin Han Kang

**Affiliations:** 1 Department of Pediatrics, Seoul St. Mary’s Hospital, College of Medicine, The Catholic University of Korea, 222 Banpo-daero, Seocho-gu, Seoul, 06591 Republic of Korea; 2 The Vaccine Bio Research Institute, College of Medicine, The Catholic University of Korea, Seoul, Republic of Korea; 3 Research Center, Green Cross Corporation, Yongin, Republic of Korea

**Keywords:** Diphtheria-tetanus-acellular pertussis vaccine, Immunogenicity, Efficacy, Safety, Mice

## Abstract

**Background:**

Tetanus-reduced dose diphtheria-acellular pertussis (Tdap) vaccination during adolescence was introduced in response to the resurgence of pertussis in various countries. A new Tdap vaccine was manufactured in Korea as a countermeasure against a predicted Tdap vaccine shortage. This study was performed to evaluate the immunogenicity, safety, and protection efficacy against *Bordetella pertussis* of the new Tdap vaccine in a murine model.

**Methods:**

Four-week-old BABL/c mice were used for assessment of immunogenicity and protection efficacy. A single dose of primary diphtheria-tetanus-acellular pertussis (DTaP) vaccine was administered, followed by a single dose of Tdap booster vaccine after a 12-week interval. Anti-pertussis toxin (PT), anti-filamentous hemagglutinin (FHA), and anti-pertactin (PRN) IgG titers were measured before primary vaccination, and before and after booster vaccination. An intranasal challenge test was performed after booster vaccination to determine protection efficacy. To assess safety, mouse weight gain test and leukocytosis promotion test were performed using 4-week-old ddY female mice.

**Results:**

Anti-PT and anti-FHA IgG titers after booster vaccination were significantly higher than those before booster vaccination with either the new vaccine or a commercially available Tdap vaccine (*P* = 0.01 for all occasions). After booster vaccination, no significant difference was observed between the two vaccines in antibody titers against pertussis antigens (*P* = 0.53 for anti-PT IgG, *P* = 0.91 for anti-FHA IgG, *P* = 0.39 for anti-PRN IgG). In the intranasal challenge test, inoculated *B. pertussis* was eradicated 7 days after infection. On days 4 and 7 after infection, colony counts of *B. pertussis* were not significantly different between the new and positive control vaccine groups (*P* = 1.00). Mean body weight changes and leukocyte counts of the new vaccine, positive control, and negative control groups were not significantly different 7 days after vaccination (*P* = 0.87 and *P* = 0.37, respectively). All leukocyte counts in the new vaccine group were within a mean ± 3 standard deviations range.

**Conclusions:**

A murine model involving a single dose primary DTaP vaccination followed by a single dose Tdap booster vaccination can be used for non-clinical studies of Tdap vaccines. The new Tdap vaccine manufactured in Korea exhibited comparable immunogenicity, protection efficacy, and safety with a commercially available Tdap vaccine.

## Background

*Bordetella pertussis* is the causative agent of pertussis, which is characterized by a paroxysmal cough, and may lead to severe complications and mortality [1, 2]. Occurrence of pertussis has decreased since the introduction of the diphtheria-tetanus-whole cell pertussis (DTwP) vaccine in the 1950s, which contains pertussis antigens as well as diphtheria and tetanus toxoids [1]. The diphtheria-tetanus-acellular pertussis (DTaP) vaccine was later developed to reduce the severe adverse effects associated with pertussis antigens included in the DTwP vaccine [3]. In Korea, DTaP vaccines have replaced DTwP vaccines since 1985 [4], and in other western countries, DTaP vaccines have been administered since the 1990s [1, 5]. However, resurgence of pertussis was observed in various western countries after then [1, 2, 5], and it has also been observed in Korea since the 2000s [4]. Such resurgence of pertussis is believed to be a consequence of waning immunity against pertussis acquired by DTaP vaccination, and therefore, a booster vaccination with a tetanus-reduced dose diphtheria-acellular pertussis (Tdap) vaccine during adolescence was introduced [6, 7]. In anticipation of a predicted Tdap vaccine shortage, the Green Cross Corporation (GCC; Yongin, Korea), a pharmaceutical company of Korea, developed a new Tdap vaccine [8].

The World Health Organization (WHO) recommends conducting non-clinical trials for newly developed vaccines containing acellular pertussis (aP) antigens, if the vaccine contains a novel antigen or is manufactured by a new manufacturer, new process, or new strain [9]. We previously reported the immunogenicity and protection efficacy of the newly developed GCC Tdap vaccine in a murine model in 2015, and demonstrated that the new vaccine showed comparable efficacy with a commercially available Tdap vaccine [8]. However, in the previous study, a robust antibody response was observed after two doses of primary DTaP vaccination, which prevented differentiation of the effects of the Tdap booster vaccine [8].

The present study was performed to evaluate the immunogenicity, protection efficacy, and safety of the new GCC Tdap vaccine in a murine model, using a strategy that addressed the limitations of the previous study. This study will assist in the establishment of future non-clinical trials on Tdap vaccines.

## Methods

### Assessment of immunogenicity

Four-week-old BALB/c female mice were acquired from Orientbio Co. Ltd. (Seongnam, Republic of Korea), and the mice were housed under semi-specific pathogen-free conditions with food and water available ad libitum. A single dose of DTaP vaccine was administered as the primary vaccination, and Tdap booster vaccination was performed 12 weeks later. The mice were divided into five groups according to the primary and booster vaccines administered (Table 1). Group 1 mice were injected with phosphate-buffered saline (PBS) as both primary and booster vaccines, and Group 2 mice were administered with DTaP vaccine (Infanrix®, GlaxoSmithKlein, Middlesex, UK) as the primary vaccination and PBS as the booster vaccination. Primary DTaP vaccination and booster tetanus-reduced dose diphtheria (Td) vaccination were administered to mice in Group 3. Those in Groups 4 and 5 received primary DTaP vaccination, and either the new GCC Tdap vaccine (Group 4) or a commercially available Tdap vaccine (Boostrix®, GlaxoSmithKlein; Group 5) as the booster vaccination. The GCC Tdap vaccine and Boostrix® contained identical doses of pertussis antigens: pertussis toxin (PT) 8 μg, filamentous hemagglutinin (FHA) 8 μg and pertactin (PRN) 2.5 μg within 0.5 mL. One-fourth the human dose (0.125 mL) was injected intraperitoneally for all vaccines and PBS as like previous murine model studies on the immunogenicity of aP vaccines [10–12].

**Table 1:** Characteristics of study groups

Group	Primary vaccine	Booster vaccine
Group 1	Phosphate-buffered saline	Phosphate-buffered saline
Group 2	DTaP	Phosphate-buffered saline
Group 3	DTaP	Td
Group 4	DTaP	Tdap (new GCC vaccine)
Group 5	DTaP	Tdap (positive control vaccine)

*DTaP* diphtheria-tetanus-acellular pertussis, *GCC* Green Cross Corporation, *Td* tetanus-reduced dose diphtheria, *Tdap* tetanus-reduced dose diphtheria-acellular pertussis.

The immunogenicity of vaccines was evaluated by measuring antibody titers against three pertussis antigens (anti-PT IgG, anti-FHA IgG and anti-PRN IgG) using commercially available enzyme-linked immunosorbent assay (ELISA) kits (Alpha Diagnostic International Inc., San Antonio, TX, USA). Blood samples were collected from the retro-bulbar venous plexuses of 10 mice in each group before primary vaccination, immediately before booster vaccination, and 3 weeks after booster vaccination. Anti-diphtheria toxoid (DT) IgG and anti-tetanus toxoid (TT) IgG titers were also measured using commercially available ELISA kits (Alpha Diagnostic International Inc.). Antibody titers of each tested antigen were compared between mice groups at each timepoint, and titers before and after booster vaccination were compared within each group.

### Intranasal challenge test

The protection efficacy against *B. pertussis* infection was evaluated using an intranasal challenge test in Groups 2, 4, and 5. A *B. pertussis* strain obtained from a Korean adult pertussis patient, which was supplied from the Korean Centers for Disease Control & Prevention (No. 13674), was intranasally inoculated at 6 × 10^6^ colony forming units (CFUs) 4 weeks after booster vaccination. Considering that the emergence of genetically mutated *B. pertussis* strains escaping from vaccine effects was proposed to be a cause of resurgence of pertussis in recent years [13], the vaccine effect on the *B. pertussis* strain isolated from a pertussis patient rather than vaccine strain could be more reliable in the real world. Five mice in each group were sacrificed and their lungs were extracted 2 h, 2 days, 4 days, 7 days, and 9 days after intranasal infection. The lungs were homogenized in 10 mL of PBS, and the homogenates were diluted to concentrations of 10^−1^, 10^−3^, and 10^−5^. Each diluted homogenate was incubated on a Bordet-Gengou agar plate at 36 °C for 4 days. CFUs of cultured *B. pertussis* on each agar plate were then determined, and mean CFUs were compared between mice groups at each timepoint.

### Assessment of safety

Mouse weight gain test (MWGT) and leukocytosis promotion (LP) test were performed to determine the safety of the GCC Tdap vaccine. Four-week-old ddY female mice (Orientbio Co. Ltd.), which were used in a previous study on the safety assessment of aP vaccines [14], were also used in the present study. Three groups of 10 mice were intraperitoneally inoculated with the GCC Tdap vaccine (test group), a commercially available Tdap vaccine (Boostrix®, positive control group), and PBS (negative control group), respectively. Each vaccine was administered at 25% of the human dose (0.125 mL). The mice were weighed before vaccination and 1, 2, 5, 6, and 7 days after vaccination. The mean body weights of the three groups were compared at each timepoint, and the vaccine was considered to be non-toxic if the mean body weight gain of the vaccinated group exceeded 60% of that of the negative control group 7 days after vaccination.

For the LP test, leukocyte counts were determined from tail vein blood samples collected 7 days after vaccination. Because the exact pass criteria for the LP test have not been established, leukocyte counts were simply compared between groups, and test group values were assessed to determine whether they fell consistently within the range of mean ± 3 standard deviations (SDs) for assessing the assay validity [15].

### Statistical analysis

Antibody titers in Groups 1 to 5 were compared using a Kruskal-Wallis test at each sampling timepoint. To assess immunogenicity for each group, antibody titers before and after booster vaccination were compared using a Wilcoxon signed rank test. For the intranasal challenge test, CFU values of *B. pertussis* in the lungs of GCC vaccinated and positive and negative control mice were compared using a Kruskal-Wallis test at each timepoint. For the safety assessment, mean body weights at each timepoint and leukocyte counts 7 days after vaccination were compared between groups using a one-way analysis of variance test. Statistical analysis was performed using SPSS Statistics version 18.0 (SPSS Inc., Chicago, IL, USA), and statistical significance was defined as a two-tailed *P* value<0.05.

## Results

### Immunogenicity of the Tdap vaccine

**Fig. 1:**
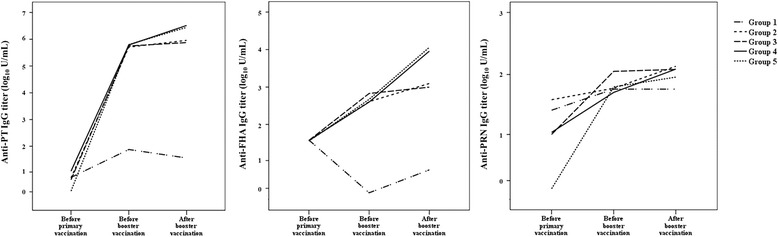
Tdap booster vaccine immunogenicity determined by titers of antibodies against pertussis antigens before and after vaccination

IgG titers against pertussis antigens before primary vaccination were not significantly different among the five groups (*P* = 0.07 for anti-PT IgG, *P* = 0.57 for anti-FHA IgG, *P* = 0.91 for anti-PRN IgG, Fig. 1). Prior to booster vaccination, anti-PT IgG (*P* < 0.01) and anti-FHA IgG (*P* < 0.01) titers in Group 1 were significantly lower than all other groups. After booster vaccination, anti-PT IgG and anti-FHA IgG titers significantly increased in Group 4 (*P* = 0.01, *P* = 0.01, respectively) and Group 5 (*P* = 0.01, *P* = 0.01, respectively). However, no significant differences were observed in anti-PRN IgG titers before and after booster vaccination in these groups. There was no significant difference in anti-PT IgG, anti-FHA IgG, and anti-PRN IgG titers between the Groups 4 and 5 after booster vaccination (Fig. 1).

**Fig. 2:**
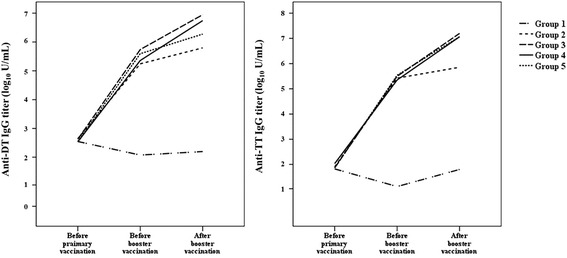
Tdap booster vaccine immunogenicity determined by titers of antibodies against diphtheria and tetanus toxoids before and after vaccination

Anti-DT IgG (*P* = 0.54) and anti-TT IgG (*P* = 0.41) titers were not significantly different among the five groups before primary vaccination, and titers in Groups 2 to 5 were significantly higher than that in Group 1 after primary DTaP vaccination (*P <* 0.01, *P* = 0.01, respectively, Fig. 2). After booster vaccination, anti-DT IgG (*P* < 0.01) and anti-TT IgG (*P* < 0.01) titers in Groups 3 to 5 were significantly higher than in Groups 1 and 2. Among Groups 3 to 5, anti-TT IgG titers after booster vaccination were not significantly different (*P* = 0.34); however, the anti-DT IgG titer in Group 3 was significantly higher than in Groups 4 and 5 after booster vaccination (*P* = 0.04).

### Protection efficacy of the Tdap vaccine

**Fig. 3:**
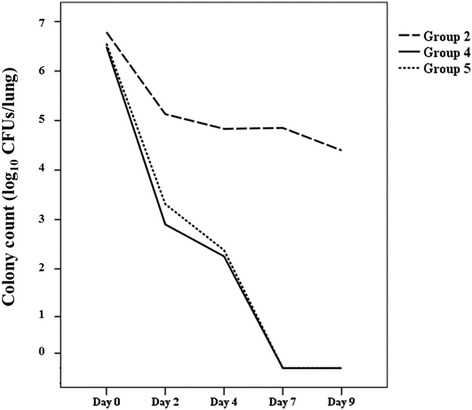
Tdap booster vaccine protection efficacy determined by lung *Bordetella pertussis* colony counts after intranasal challenge test

On the day of intranasal challenge, colony counts of *B. pertussis* in the lungs were not significantly different between Groups 2, 4, and 5 (Fig. 3). However, on days 2, 4, and 7 after intranasal challenge, CFUs of Groups 4 and 5 were significantly lower than that of Group 2 (*P* < 0.01, *P* = 0.01, *P <* 0.01, respectively). Colony counts 2 days after infection showed a significant difference between Groups 4 and 5 (*P* = 0.02); however, no significant differences were observed at any subsequent timepoints.

### Safety of the Tdap vaccine

**Fig. 4:**
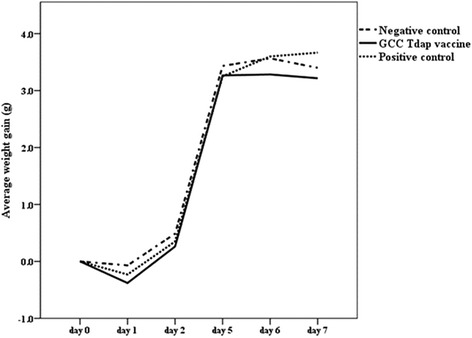
Tdap booster vaccine safety assessed by mouse weight gain test

Mean body weights of the GCC vaccinated, positive control and negative control groups showed no significant differences prior to vaccination, and mean body weight changes assessed 1, 2, 5, 6, and 7 days after vaccination showed no significant differences between groups (Fig. 4). Seven days after vaccination, mean body weight changes of the GCC vaccinated, positive control and negative control groups were 3.2 ± 0.7 g, 3.7 ± 1.7 g and 3.4 ± 0.8 g, respectively (*P* = 0.87). The mean body weight change of the GCC vaccinated group was therefore greater than 60% of those of the positive and negative control groups.

The mean leukocyte counts 7 days after vaccination of the GCC vaccinated, positive control and negative control groups were 5630 ± 1542/μL, 6495 ± 1627/μL, and 5385 ± 1497/μL, respectively, and showed no significant differences between the three groups (*P* = 0.37). The range of leukocyte counts in the GCC vaccinated group was 4450/μL to 9850/μL, and all values were within a mean ± 3 SD range.

## Discussion

In the present study, the immunogenicity and safety of a newly developed Tdap vaccine were determined in a murine model. The new Tdap vaccine showed comparable immunogenicity, protection efficacy, and safety with a commercially available Tdap vaccine.

The WHO recommends a non-clinical study as well as a clinical study before approval and implementation of a new vaccine containing aP antigens [9]. Accordingly, several non-clinical trials on the immunogenicity, protection efficacy, and safety of DTaP vaccines have been reported [10, 11, 16, 17]. However, only a few non-clinical trials on Tdap vaccines have been reported as commercially available Tdap vaccines were manufactured using the same aP antigens included in approved DTaP vaccines [12], and Tdap vaccines were approved based on clinical trial results [18–20]. Although non-clinical trials are necessary for Tdap vaccines manufactured by companies that have not previously produced DTaP vaccines, the methodology of these trials has yet to be established. In particular, it is essential to determine whether Tdap vaccines containing lower doses of diphtheria toxoid and pertussis antigens compared with DTaP vaccines can induce a boosting effect. We have previously reported preliminary results from a non-clinical study of the new GCC Tdap vaccine using a murine model [8]. In this study, mice received two doses of DTaP vaccine as primary vaccination followed by a single dose of Tdap vaccine as booster vaccination, a method that considered the human vaccination schedule of four doses of DTaP vaccine and one dose of Tdap vaccine [8]. Although the new Tdap vaccine showed favorable immunogenicity and protection efficacy against *B. pertussis* intranasal challenge, antibody titers against pertussis antigens were high even before Tdap booster vaccination, and inoculated *B. pertussis* was completely eradicated within 5 days of intranasal challenge [8]. These results are believed to be a consequence of the prolonged effect of two shots of DTaP vaccination supplementing the boosting effect of Tdap vaccination. As a result, primary DTaP vaccination was administered as a single dose in the present study, and the interval between the primary and booster vaccinations was extended from 6 weeks to 12 weeks.

In the present study, anti-PT IgG and anti-FHA IgG titers significantly increased in the GCC vaccine group (Group 4) and positive control group (Group 5) after Tdap booster vaccination, and no significant difference was observed after booster vaccination between the two groups. Therefore, the immunogenicity of a Tdap vaccine can be determined using one dose of primary DTaP vaccine followed by one dose of Tdap booster vaccine in a murine model, and the new GCC Tdap vaccine showed immunogenicity comparable with a commercially available Tdap vaccine using this strategy. In a previous non-clinical study of a Tdap vaccine in a murine model, one dose of primary DTwP vaccine was administered followed by one dose of Tdap booster vaccine 12 weeks later [12]. The boosting effect of the Tdap vaccine decreased upon reduction of pertussis antigen dose in the primary DTwP vaccine, and the authors therefore recommended an increased interval between primary and booster vaccinations to rule out residual effects of primary vaccination [12]. In the present study, the antibody titers against pertussis antigens, DT and TT tended to increase after booster vaccination even in the Groups 2 and 3, in which PBS or Td vaccine was administered as the booster vaccination, although the anti-PT and anti-FHA IgG titers against pertussis antigens in the Groups 2 and 3 were lower than those in the Groups 4 and 5. This result could be caused by the prolonged effects of the primary DTaP vaccination in the Groups 2 and 3. Therefore, the interval between primary and booster vaccinations should be longer than 12 weeks to completely exclude the residual effects of the primary vaccination.

An intranasal challenge test was additionally performed to determine the protection efficacy of the Tdap vaccine against *B. pertussis* in the present study, although the WHO recommendations for non-clinical studies only specify antibody tests [9]. Because *B. pertussis* was eradicated within 5 days of intranasal infection in the preliminary study [8], colony counts were determined 2 and 4 days after infection in the present study. With the use of a single dose of DTaP vaccine, the bacteria were eradicated 7 days after intranasal infection, 2 days later than with two doses. The GCC vaccine group showed significantly lower CFUs than the positive control group 2 days after infection, indicating a potent effect in the early phase. If we consider that the results of the intranasal challenge test in a murine model are representative of clinical efficacy in humans [11], the new GCC Tdap vaccine may be used effectively in the clinical field. In addition, the new Tdap vaccine showed comparable results with a commercially available Tdap vaccine in MWGT and LP test. Therefore, further clinical trials for the new Tdap vaccine may be performed safely. In mice treated with DTaP vaccine at 25% of the human dose, inoculated *B. pertussis* persisted longer than 9 days after intranasal challenge in the negative control group injected with normal saline as a booster vaccination. We thought this dose was sufficiently low to prevent residual effects of the primary DTaP vaccination on the boosting response of the Tdap vaccine, given the 12-week interval between primary and booster vaccinations; however, an interval longer than 12 weeks could be appropriate to exclude the residual effects, considering the results of the immunogenicity assessment.

This study has several limitations. Neither the GCC nor the commercial Tdap vaccine had a significant effect on anti-PRN IgG titers. However, significant humoral immune responses to PT and FHA and a significant protection efficacy in the intranasal challenge model were observed in both groups. Although cellular immune responses also play an important role in the protective immunity against *B. pertussis* [17, 21–23], they were not measured in the present study. In previous studies, Th1 and Th17 responses were detected after DTwP vaccination and natural *B. pertussis* infection, and Th2 responses were detected after DTaP vaccination [17, 21, 22, 24]. In contrast, some investigators reported Th1-dominant responses or Th1 and Th2 mixed responses even after DTaP vaccination [16, 25]. Moreover, cellular immune responses after booster DTaP or Tdap vaccination differed according to the type of primary vaccination in some studies [12, 26]. Therefore, future studies should determine cellular immune responses elicited by Tdap booster vaccination. Based on the results of cellular immune responses of the new Tdap vaccine with a combination of the results of the present study, clinical trials should be planned. For in vivo assessment of residual toxicity of aP antigens, a histamine sensitization test is recommended [9]. The present study instead used MWGT and LP test. The histamine sensitization test is supposed to be performed using a GCC DTaP vaccine containing an increased aP antigen dose than the GCC Tdap vaccine.

## Conclusions

In conclusion, a murine model consisting of a single dose of primary DTaP vaccination followed by a single dose of booster Tdap vaccination can be applied to non-clinical studies of Tdap vaccines with an interval longer than 12 weeks between primary and booster vaccinations. When tested according to this model, the new GCC Tdap vaccine exhibited comparable immunogenicity, protection efficacy, and safety with a commercially available Tdap vaccine. Future clinical trials using the new GCC Tdap vaccine can be performed after confirmation of appropriate cellular immune responses in non-clinical trials.

## Abbreviations

CFU: Colony forming unit
DT: Diphtheria toxoid
DTaP: Diphtheria-tetanus-acellular pertussis
DTwP: Diphtheria-tetanus-whole cell pertussis
FHA: Filamentous hemagglutinin
GCC: Green Cross Corporation
LP: Leukocytosis promotion
MWGT: Mouse weight gain test
PBS: Phosphate-buffered saline
PRN: Pertactin
PT: Pertussis toxin
SD: Standard deviation
Td: Tetanus-reduced dose diphtheria
Tdap: Tetanus-reduced dose diphtheria-acellular pertussis
TT: Tetanus toxoid
WHO: World Health Organization
